# Clinical Application of Next-Generation Sequencing as A Liquid Biopsy Technique in Advanced Colorectal Cancer: A Trick or A Treat?

**DOI:** 10.3390/cancers11101573

**Published:** 2019-10-16

**Authors:** Myrto Kastrisiou, George Zarkavelis, George Pentheroudakis, Angeliki Magklara

**Affiliations:** 1 Laboratory of Clinical Chemistry, Faculty of Medicine, School of Health Sciences, University of Ioannina, 45110 Ioannina, Greece; myrto.kastrisiou@gmail.com; 2 Department of Medical Oncology, University General Hospital of Ioannina, 45500 Ioannina, Greece; gzarkavelis@outlook.com (G.Z.); gpenther@otenet.gr (G.P.); 3 Society for Study of Clonal Heterogeneity of Neoplasia (EMEKEN), 45444 Ioannina, Greece; 4 Department of Biomedical Research, Institute of Molecular Biology & Biotechnology, Foundation for Research & Technology-Hellas, 45110 Ioannina, Greece

**Keywords:** next-generation sequencing, colorectal cancer, circulating tumor DNA, liquid biopsies, cell-free DNA

## Abstract

Owing to its advantages over prior relevant technologies, massive parallel or next-generation sequencing (NGS) is rapidly evolving, with growing applications in a wide range of human diseases. The burst in actionable molecular alterations in many cancer types advocates for the practicality of using NGS in the clinical setting, as it permits the parallel characterization of multiple genes in a cost- and time-effective way, starting from low-input DNA. In advanced clinical practice, the oncological management of colorectal cancer requires prior knowledge of *KRAS*, *NRAS*, and *BRAF* status, for the design of appropriate therapeutic strategies, with more gene mutations still surfacing as potential biomarkers. Tumor heterogeneity, as well as the need for serial gene profiling due to tumor evolution and the emergence of novel genetic alterations, have promoted the use of liquid biopsies—especially in the form of circulating tumor DNA (ctDNA)—as a promising alternative to tissue molecular analysis. This review discusses recent studies that have used plasma NGS in advanced colorectal cancer and summarizes the clinical applications, as well as the technical challenges involved in adopting this technique in a clinically beneficial oncological practice.

## 1. Introduction

Colorectal cancer (CRC) is the third most common and second most lethal neoplasm, according to the latest cancer statistics [1]. Classic cytotoxic chemotherapy is still the backbone of many therapeutic regimens in modern oncology, including CRC cases. However, over the past decade, there has been a shift towards personalized medicine, and current research mainly focuses on identifying possible actionable mutations [2]. In addition, immunotherapy has provided a significant overall survival gain in cancer patients, and is being applied based on molecular biomarkers indicating a highly immunogenic tumor environment as a result of the tumor’s mutational burden and profile [3].

Current guidelines suggest that specific molecular biomarkers should be examined before the initiation of a patient’s therapy in several neoplasms, thus paving the way towards the practice of “picking the target” in each patient [2]. As the number of mutations with clinical relevance is gradually expanding, the need for multiplex tumor genomic profiling is now widely recognized. In this light, next-generation sequencing (NGS) is gaining ground over the “gold standard” Sanger sequencing, the latter being limited by its lower sensitivity, scalability, and cost-effectiveness for the molecular characterization of tumors [4].

Liquid biopsies are emerging as possible alternatives to classic tissue biopsy, providing the molecular signature of tumors with a single blood aspiration and without the need of applying invasive techniques [5]. Circulating tumor DNA (ctDNA) is a liquid biopsy biomarker that provides information for the genetic alterations of the tumor, and it has shown great promise in the clinical setting [6]. The use of NGS as a method for the analysis of ctDNA in CRC cases may provide valuable insight into tumor heterogeneity and clonal evolution, and it may reveal novel molecular targets for the application of individualized therapy [7,8].

The aim of the present review is to provide a concise picture of the clinical studies that have been performed using NGS for the analysis of ctDNA in metastatic CRC (mCRC). We chose to focus on the technical parameters of NGS, which are often overlooked by clinicians who are asked to interpret plasma NGS results, as well as on clinical aspects, often missed by assay developers and laboratory scientists performing NGS analyses. The reader is also referred to other recent reviews in the field that report on various methodologies of ctDNA detection and its clinical utility in cancer [9], and discuss the application of evidence-based precision medicine in the disease [10].

## 2. Background

### 2.1. Colorectal Cancer: From Molecular Heterogeneity to Optimal Therapy Selection

In 25% of CRC cases, the cancer has already spread to distant organs at diagnosis, and another 50% of patients will develop metastatic disease later on [1,2]. The initial treatment of early-stage CRC is surgical excision. According to the results of the pathology report, clinicians decide whether to administer cytotoxic chemotherapy. However, in more advanced cases of CRC where surgery is not an option, patients are offered chemotherapy, with life prolongation and symptoms alleviation being the main goals. In selected cases, efforts are made to achieve cytoreduction permissive to surgical excision that would render the patient disease-free [2]. With the advent of targeted therapies, the face of cancer treatment has changed. In mCRC, current guidelines require *KRAS* and *NRAS* exons 2, 3, and 4, as well as *BRAF* exon 15 profiling, as a prerequisite for optimal therapy selection [11]. Likewise, *ERBB2* and *PIK3CA* gene mutations and *NTRK* gene fusions constitute “emerging biomarkers” studied in many clinical trials [12,13,14,15].

In the past decade, CRC therapeutics were marked by the introduction of anti-EGFR monoclonal antibodies and antiangiogenetic factors for the treatment of metastatic disease [2]. The administration of such targeted agents along with cytotoxic medication has shown prolongation of the median overall survival of patients to up to 30 months, higher response rates, and progression-free survival, depending upon the mutational profile of CRC [2]. In mCRC, as in most other neoplasms, the emergence of secondary resistance is a common problem involving targeted therapies. Resistance usually appears a few months after the onset of therapy. Among the driving mechanisms that lead to treatment failure, the positive selection of cancer clones with inherent or acquired anti-EGFR resistance potential prevails [16]. According to published results, *KRAS*, *NRAS*, *BRAF*, *HER2*, *MET*, *PIK3CA*, *PTEN*, and *MAPK* mutations, among others, are the most common molecular events that drive resistance to anti-EGFR therapy [16,17]. Tumor profiling revealing the status of these genes at the time of treatment failure would offer substantial insight into such molecular events, and would also provide a priori knowledge of the forthcoming resistance to therapy and patients’ clinical deterioration. Even if re-biopsy of the primary tumor or metastasis was deemed safe and feasible, it would not account for the tumoral heterogeneity and would underrepresent other disease sites, possibly containing resistant clones [18]. In this setting, utilizing a liquid biopsy approach is very appealing, allowing rapid detection of acquired resistance and for treatment to be adapted accordingly [18]. Furthermore, data support the notion that patients who develop resistance to anti-EGFR antibodies might be able to re-sensitize after a period of anti-EGFR therapy withdrawal [17]. Following an anti-EGFR therapy “holiday”, CRC cells are considered to repopulate, making the tumor once again sensitive to anti-EGFR treatment [16,17]. In this light, identification of *KRAS* and *NRAS* mutations with longitudinal liquid biopsies is expected to be requested by clinicians at later lines of mCRC treatment as well, with both prognostic and predictive potential.

The molecular classification of neoplasms has become of interest to the medical oncology community, due to its potential in the successful practice of personalized medicine. The four molecular subtypes identified in CRC provide prognostic and other clinical information and, as of late, they also offer some predictive information for the application of immunotherapy in hypermutated tumors [19]. The application of NGS in liquid biopsies can be used for the molecular classification of colorectal tumors, aiding in the prognosis and the choice of therapeutic strategies.

### 2.2. Circulating Tumor DNA as A Promising CRC Biomarker

The detection of circulating tumor DNA (ctDNA) in blood has emerged as a promising alternative to tissue molecular profiling, and is recognized as the liquid biopsy biomarker with the most clinical applications [20]. Cancer cells are known to release genetic material in the blood circulation that can be isolated from patients’ plasma and allow for identification of the neoplastic molecular signature [8,21]. Ranging from 0.005–85% [21], ctDNA typically constitutes <1% in limited amounts of cell-free DNA (cfDNA) in the blood [22] and its detection can often be very challenging. However, ctDNA-based liquid biopsies can overcome several drawbacks that are inherent to conventional biopsies. A single blood aspiration is simple and easy without the events of infection, hemorrhage, wounds, and complications that accompany invasive biopsies [8]. Furthermore, the detection of ctDNA provides real-time access to the genetic information and allows for serial monitoring of the patient, contrary to tissue biopsy that only depicts the molecular profile of the tumor in the specimen examined [7,16,17].

As described above, CRC is a heterogenous disease characterized by intratumoral multiclonality, diversity of mutations among metastases and acquired mutations in time, and this molecular complexity ultimately leads to resistance to the administered therapeutic regimen. The use of ctDNA provides insight into this molecular diversity, and grants the necessary information for diagnosis, therapy selection, minimal residual disease, and patient follow-up [7,16,23]. CRC is among the neoplasms where ctDNA can be identified, especially in the presence of metastatic disease. In earlier stages of the disease, ctDNA can be identified in approximately 50% of cases, but this number exceeds 90% when there is extensive tumor load, making it a reliable biomarker and source of genetic information for CRC patients [7].

After a curative surgical excision of a local or locally advanced CRC, the patient is considered to be “free of disease” provided that the pathology report states that the surgical excision was complete and that the surgical margins are clear. However, detection of ctDNA in these patients post resection indicates a higher probability of disease relapse in the foreseeable future [23]. The clinician can thus become aware of this possibility and closely monitor the patient. In addition, apart from elucidating mechanisms of resistance, the identification of newly acquired mutations in ctDNA may lead to the implementation of new targeted therapies, expanding the armory against CRC. Ongoing clinical studies utilize the detection of ctDNA in blood circulation as a liquid biopsy method to guide the administration of targeted agents according to the mutations identified in relapsing CRC patients [24]. Furthermore, ctDNA can be detected in bodily fluids other than blood, such as urine, cerebrospinal fluid, and pleural effusion [25]. The detection of ctDNA in urine samples from CRC patients using NGS has been associated with tumor load, while the comparison between tumor tissue and urine mutant *KRAS* was highly concordant [26].

In conclusion, minimally invasive techniques can be implemented for the detection and monitoring of ctDNA in CRC, which serves as a reliable “proxy” of the molecular make-up of all tumor sites in real time [27,28]. Liquid biopsies have been extensively validated in CRC through clinical trials but they have so far not gained approval for any clinical implementation. More robust results are still needed for the integration of ctDNA as a clinical biomarker in optimal therapy selection, emergence of resistance, and molecular classification. However, already published data advocate that it will fulfil its promise in the near future [29]. On the other hand, the application of ctDNA for CRC diagnosis remains elusive, as it cannot bypass the sensitivity and specificity issues of traditional biomarkers [30].

### 2.3. Next-Generation Sequencing in CRC

As previously stated, the need for the detection of multiple genetic aberrations (for the treatment of human cancers) in a cost-effective and time-saving manner has led to the implementation of massively parallel sequencing technologies known as next-generation sequencing (NGS) in translational cancer research. Leaving behind the first generation of Sanger sequencing, NGS emerged with great potential and found applications in various fields, including basic cancer research and medical oncology [31,32]. Whole-genome sequencing (WGS) covers all regions of one’s genetic material, in contrast with whole-exome sequencing (WES), which only covers the coding parts of DNA. Due to the high cost and turnaround time of both WGS and WES, the far more viable alternative of targeted NGS, which only covers genomic regions of interest, has been widely adopted in both research and clinical practice [33,34]. In cancer medicine, the focus is on cancer gene “hotspots”, where recurrent mutations occur. Targeted testing of what is known as a gene panel allows for greater sensitivity, and is therefore very practical in the molecular diagnosis of even very rare molecular aberrations [35].

The first step in targeted sequencing is the enrichment of regions of interest via PCR amplification or hybrid capture strategies. While the former is known to require shorter preparation time and a lower amount of input DNA, the latter is generally believed to have higher accuracy [36]. However, significant advances in PCR amplification or amplicon-based assays, including the use of unique molecular identifiers, have rendered them a reliable alternative for targeted enrichment, or NGS library preparation [37,38].

Regarding the application of NGS in CRC, initial studies were aimed at evaluating the use of this technology in tissue biopsy to identify possible mutations that correlated with CRC prognosis or appropriate therapy selection [39]. The overall conclusion of those studies was that this was a valid technique for the identification of hotspot gene mutations such as *KRAS*, *NRAS*, and *BRAF*, that were a prerequisite for the selection of treatment of mCRC patients. Furthermore, it became evident that the application of massive parallel sequencing allowed for identification of additional mutations in the patient tumor samples, thus providing insight into the heterogeneity of the disease [28].

More recent studies have interrogated the use of NGS in the detection of liquid biopsy biomarkers in CRC, and they are discussed in this review. Specifically, our review aims to present the data from the clinical application of plasma NGS in mCRC in a coherent and succinct manner, a task that to our knowledge has not been undertaken before.

Many next-generation sequencers are currently on the market, but it is beyond our aim to analyze each technology, and the reader is referred to other excellent reviews on the subject [35,40,41]. In the studies described below, Illumina and Life Technologies sequencers were almost equally used. The former, including the MiSeq, HiSeq, and NextSeq models, are based on sequencing by synthesis with the incorporation of reversible dye-terminators that emit unique optical signals [42]. The latter, namely Ion Proton, Ion PGM, and Ion S5, measure changes in voltage following the release of a hydrogen ion during the polymerization of DNA (“semiconductor sequencing”) [42]. The more recently launched Guardant360 digital sequencing technology, also used in a number of studies, has been strategically developed solely as a liquid assay since the beginning [43].

## 3. Clinical Application of ctDNA NGS in Advanced CRC Patients

### 3.1. Review Methods

Two independent reviewers searched the PubMed database for English-language studies on the use of NGS as a liquid biopsy approach in CRC. The search algorithm employed contained the key words “next generation sequencing or NGS” and “colorectal cancer”, as well as one of the following: “liquid biops*”, “ctDNA” and “cfDNA”. Definition of the clinical stage was not included as a keyword in order to avoid the exclusion of studies with early- as well as late-stage CRCs. Studies including only stages I–III were manually excluded by the reviewers as not relevant, as were studies not clarifying the stage of CRC or not presenting a distinct mCRC cohort.

**Figure 1:**
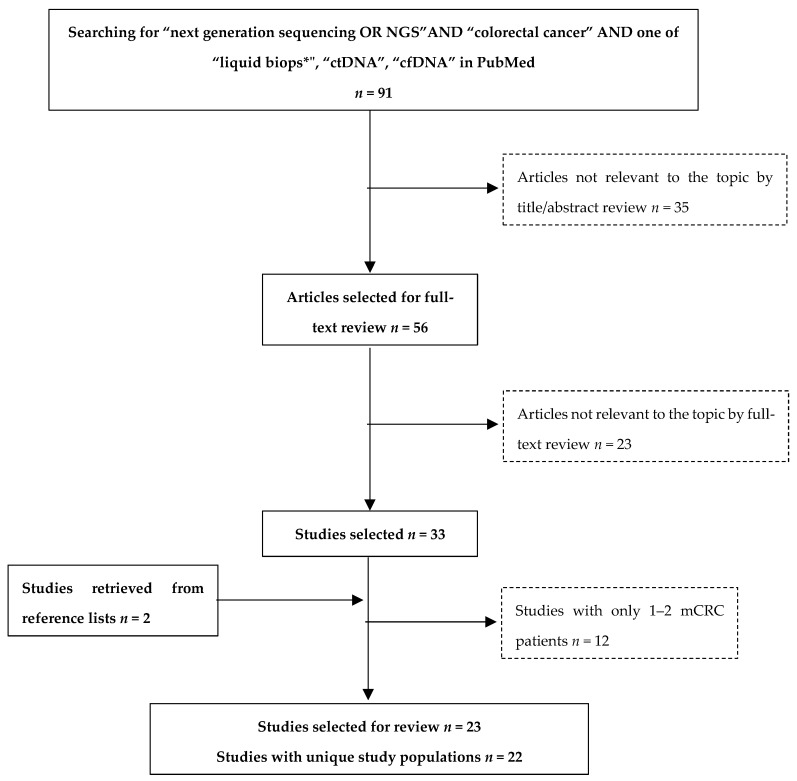
Study selection process.

The study selection process that was followed is depicted in Figure 1. Ninety-one publications were initially retrieved and 56 were deemed eligible based on title/abstract review. Upon full-text review, 23 publications were excluded for the following reasons: (a) two were reviews, one was a book chapter, and another one was a comment on another publication; (b) one study employed whole-exome sequencing, while another three did not perform any NGS-based plasma testing; (c) fifteen studies were not found to report any relevant information to our review. In order to minimize publication bias, we further screened the reference lists of the above studies. Thus, two more publications were retrieved [44,45,46]. For the sake of data comparability, we opted to present clinical data from studies with a satisfactory number of mCRC patients, further excluding 12 of the above publications. Four were case reports, and eight included only 1–2 patients with mCRC among other cancer types and stages. It was noted that two studies referred to the population of the ASPECCT clinical trial [47,48]. It was thus decided to present the most recent one, as it contains updated trial data.

### 3.2. Overview of Studies

A summary of eligible publications and their characteristics is provided in Table 1. Overall, 19 out of 22 publications that included unique study populations were exclusively about CRC, all of them focusing on advanced or metastatic stages. Distinct metastatic patient cohorts were also described by Rachiglio et al., Kim et al., and Onidani et al., despite the inclusion of other types of cancer (metastatic non-small-cell lung cancer (mNSCLC), other solid tumors, head and neck and other gastrointestinal tumors, respectively) [49,50,51]. Some of the CRC-specific studies also defined some treatment-related entry criteria, such as treatment with anti-VEGF or anti-EGFR agents, at the first [48,52,53] or later lines of therapy [44]. Others required patients receiving surgical treatment for liver metastases [54] or who were chemotherapy-naïve [45,55,56,57].

The range of the advanced CRC sample size was reported to be between 12 and 1397 patients for CRC-only studies, and 32–35 patients in mixed-histology studies (see Table 1). In this review, we selectively present the available data on CRC patients.

Some studies only aimed at assessing the feasibility and clinical relevance of NGS plasma assays [49,50,62,63,66,67], while several others employed NGS in serial samples to monitor response to treatment [45,46,56,57,60,68] and/or to study the development of resistance to administered therapies [44,48,51,52]. The goal of two studies was to decipher the genomic landscape of cfDNA to grasp CRC heterogeneity [59,64]. Other studies sought to compare plasma NGS with reference methods in tissue [55], or with plasma digital PCR [53,54,61]. Another group evaluated four different manual extraction procedures and the addition of various carrier molecules into the plasma (i.e., carrier RNA, polyadenylic acid, glycogen, linear acrylamide, yeast tRNA, and salmon sperm DNA) to improve ctDNA extraction recovery [58].

Table 2 provides a summary of the NGS platforms used in the reviewed studies, as well as an account of gene panels and library preparation tools used in each of them. Commercially available panels, as opposed to house-made ones, were used in 15 studies. More specifically, the Ion AmpliSeq Colon & Lung Panel v2 (22 genes) was used twice [55,61], on Ion PGM and Ion Proton sequencers, respectively. The same panel was possibly used in a third study, which refers to analysis of “hotspot and targeted regions of 22 genes”, “with an NGS-based panel that has been approved for in vitro clinical diagnostics in colon and lung cancer patients”, also on an Ion PGM [50]. On the same instrument, the Ion AmpliSeq Cancer Hotspot Panel v2 (50 genes) was used in one study [59] and the SV-CA50-ctDNA panel (50 genes) was used in another [56]. The AVENIO ctDNA Expanded Kit (77 genes), NCC Oncopanel (90 genes), and the oligo-gene SOMATIC 1 MASTRTM v2 were each used once, with NextSeq 500, HiSeq 2500, and MiSeq sequencers, respectively [44,52,58]. The PlasmaSelect-R (63 genes) was also used with an Illumina instrument, although the type is not specified [48]. Guardant360 was also described in four studies [49,63,64,68]. This platform currently supports the sequencing of 73 genes, but previously available panels of 54 and 68 genes were used by some of the groups, as shown in Table 2. Six groups developed and validated their own assays. In their studies, they used custom oligo- [57] or multi-gene panels [45,53,54,60,62] of moderate size (from 14 to 21 genes).

As shown in Table 2, only one study was limited to the currently actionable genes recommended in the ESMO guidelines for the treatment of advanced CRC [58]. The vast majority of studies covered a range of 12–90 genes. However, Demuth et al. only discussed the *KRAS* results in their paper, despite them having been generated from a multigene panel, as their study was aimed at comparison of NGS with droplet digital PCR [61]. Similarly, Yao et al. only studied the status of *KRAS*, *NRAS*, *HRAS,* and *BRAF* out of a panel of 40 genes tested [57]. In the AGEO RASANC study, the analysis of 22 genes was utilized to characterize the presence of ctDNA, but only the *RAS* genes’ status was taken into account [55]. Mutation-negative samples were further subject to digital PCR analysis for two methylated biomarkers, specific for CRC (*WIF1* and *NPY*), representing alternate markers for the presence of ctDNA [55]. Their results showed that samples that were mutation-negative but methylation-positive (i.e., false negatives, based on mutation testing) and characterized by the authors as “inconclusive ctDNA results” were more likely to occur in resected primary tumors and metastatic sites other than the liver [55]. In a more personalized approach, Tie et al. used NGS to screen each patient’s tumor for candidate mutations in 15 frequently mutated genes in mCRC, and subsequently tracked the ctDNA dynamics of a single mutation that was found present in the tumor, to assess its predictive value [45].

When it comes to library preparation protocols, also summarized in Table 2, most of the studies reported the use of the AmpliSeq Library Kit, either v.2 [54,56,59] or Plus [51], and Oncomine assays, either Solid Tumor DNA [50,61] or Colon cfDNA [62], all of which are amplicon-based enrichment approaches, as is the CRC01 kit reported by another group [66]. Several studies performed hybrid capture-based enrichment, with SureSelect [57] and Guardant assays [49,63,64,65,67]. KAPA Hyper-prep [52] and a custom library protocol [44] were also reported by one study each, while the rest of the studies omitted any reference to library preparation [45,48,53,55,58,60].

### 3.3. Pre-Analytical Parameters

The pre-analytical phase is of paramount importance for the reproducibility and clinical validity of laboratory tests, and liquid biopsy is no exception. In cfDNA isolation, the first challenge one encounters is achieving a high cfDNA yield, while preventing any contamination from blood-cell-derived DNA.

Although no specific guidelines exist regarding the possible anatomical site for blood aspiration, which can be either a peripheral or a central vein, the biological source seems to matter, with plasma being preferred over serum, due to its significantly lower levels of contamination from normal-cell DNA [69]. Furthermore, given the low concentration of ctDNA in a background of wild-type cfDNA, most researchers agree that more than 5 mL of whole blood is required for ctDNA detection [70,71]. Selecting the right type of blood collection tube is also highly important. As clotting can lead to cell disruption and the release of high amounts of genomic DNA, the presence of anticoagulants such as K_2_EDTA is generally recommended for cfDNA specimen collection [72].

Another hurdle to overcome is the possibility of white blood cell lysis, which would further dilute already scarce ctDNA with DNA released from leukocytes. The latter are generally stable when blood processing occurs within 4 h of its collection in K_2_EDTA tubes [72]. When time between blood draw and plasma preparation is expected to exceed 4 h, such as in cases when specimens are transported to another center, tubes containing cell stabilizers are recommended. In the routine application of liquid biopsies, the occurrence of white blood cell lysis is linked to red blood cell lysis or “hemolysis”, which is visually detectable owing to the release of hemoglobin. Thus, hemolyzed samples are generally considered unsuitable for ctDNA analysis, and this can be one of the few reasons for sample rejection. Consistent with this are the reported performance data by three studies, in which successful analysis was achieved in the majority of samples for Kim et al. and Demuth et al. [49,61] and in 100% of samples in Beranek et al. [58].

Following blood aspiration, plasma separation protocols ideally consist of two centrifugation steps, the first to separate plasma from the cell portion of blood and the second to remove any residual cells from plasma. It is well established that these steps should be performed as soon as possible after blood collection, and each of them should be followed by retrieval of the supernatant and transfer to a new tube. Immediately after its isolation, plasma should be aliquoted in volumes of 300 μL to 2 mL and stored in freezing temperatures of −20 °C or −80 °C that preserve the integrity of cfDNA for a period of several months to years, depending on the clinical and analytical goals [72].

Despite the critical importance of the steps that precede molecular analyses, one-third of the studies omit reference to them. Table 3 summarizes this information, where it was available. Blood collection tubes with EDTA were used in 12 studies. Another two studies used the cell-stabilizing Streck Blood Collection Tubes [55,60], while the rest of them did not report on the tube type. Similarly, only nine studies referred to the time from blood collection to further specimen processing. In eight of them, it was done within 3 h after sampling, following the recommendations for EDTA tubes provided above (see Table 3). There was, however, one study using EDTA tubes that permitted a timeframe of 24 h before centrifugation [54]. To assess the degree of cell lysis, the researchers measured in their samples’ cfDNA length patterns, which is known to differ depending on the origin of cfDNA. More specifically, cfDNA fragments from tumors have been found to be consistently shorter than those coming from healthy cells. In this study, however, length pattern analysis failed to provide obvious explanations for the presence or absence of mutations in patients with inconclusive results [54].

As Table 3 shows, two, rather than one, centrifugation steps were reported in 9 out of 13 studies, and sufficient whole blood volumes (10–30 mL) were used in 10 out of 12 studies with available information. Plasma volume for cfDNA extraction ranged from 1–8 mL in most of the studies that report this information, with the exception of Beranek et al. and Demuth et al., who used 0.75 and 0.2 mL, respectively [58,61]. More specifically, Demuth et al. used both 0.2 and 2 mL plasma samples to assess the accuracy of cfDNA extraction from minimal volumes [61]. Their results suggested that, although recovery was 15% lower in the extraction of cfDNA from the smaller volume, it could be sufficient for the detection of *KRAS* mutations in mCRC and, possibly, in other solid malignancies with high degree of ctDNA shedding [61].

Thanks to the minimally invasive nature of liquid biopsy, few parameters can undermine sample eligibility other than cfDNA abundance. A minimum DNA input is thus required for ctDNA analysis, which can vary substantially across platforms and library types. Among the studies reviewed, only nine provided this information, which are included in Table 3. As a general observation, the smaller amounts of input DNA (1.1 to 10 ng) used were with the Ion PGM sequencers [53,56,58,60], with the exception of Tie et al., who reported DNA input as low as 2–3 ng on a MiSeq instrument [45].

## 4. Overview of Studies Results

### 4.1. Prevalence of Mutations in Plasma

Table 4 displays the frequency of ctDNA mutations in the studies under review, where available. Detection rates of any mutation ranged from 72–98% in the studies that reported this information [45,55,56,60,62,68]. Accordingly, Bachet et al. referred to only 113 out of 412 patients, where no mutation was detected [55]. These discrepancies are plausible, given the differences in populations and, more importantly, in the gene panel size and content. Regarding mutations in *KRAS*, *NRAS*, and *BRAF*, whose frequencies in mCRC tumors are well established and consistent across clinical trials, one would expect their detection rate in ctDNA to be equally consistent [2]. However, their respective detection rates were 16–54%, 0–4%, and 3–7.32% of plasma samples, in the studies that reported them [45,56,58,60,62,65]. The authors reporting the lowest *RAS* mutation rates, namely, 16% for *KRAS*, no *NRAS* mutations, and 3% for *BRAF*, partially attributed them to the small sample size of their study (*n* = 32) [58], although it was not as small as others’ (see Table 1).

### 4.2. Comparison of Plasma NGS with Tissue Testing

Data comparing plasma NGS with matched tumor tissue testing are presented in Table 4. These data were retrieved by the majority of studies, with a few exceptions. In particular, studies whose patient populations were selected on the basis of tumor tissue RAS status [44,48,53] did not seek to compare plasma to tissue testing, nor did the studies by Onidani et al., Strickler et al., or Yamauchi et al. [51,52,64].

The first six studies in Table 4 used results from routine *RAS* testing with a PCR-based method as reference. These studies reported strong agreement from 77–96% [50,55,56,57,61,62]. A PCR-based method was also used for tissue testing in a seventh study [66]. Although it was not limited to *KRAS*, but also tested *NRAS*, *BRAF* and *PIK3CA*, this study reported similar concordance approaching 80%, which was even higher in pre-treatment samples (93%) [66].

In the next nine studies, matched formalin-fixed, paraffin-embedded (FFPE) tissue was subject to the same NGS panel that was used for ctDNA. However, these studies reported concordance in a heterogeneous manner. Gene-specific concordance was reported in three studies, ranging from 67–86% for *KRAS* [49,63,65], while mutation-specific concordance for *BRAF*^V600E^ was 100% in the two studies that reported it [49,65]. The use of the same sequencing platform in both tissue and plasma permits the study of concordance regarding all genes included in the panel. This was examined by four of the groups, who reported an overall concordance of 81–91% [45,58,60,67]. Across studies, the most commonly reported reasons for plasma–tissue discordance included biological (e.g., degradation of DNA from formalin-fixed, paraffin-embedded tissue over time; tumor heterogeneity; tumor burden; tumor stage; tumor aggressiveness; degree of ctDNA shedding in the bloodstream) and methodological (e.g., non-synchronous sampling, different sensitivities of methods used) parameters.

Some authors presented the sensitivity of plasma NGS, defined as its power to detect known tumor tissue variants in ctDNA. In the group of studies that used a PCR-based method for tissue testing, sensitivity was assessed in five of them and was found to range considerably (63–100%) [50,55,56,57,66]. Looking at each study separately, Rachiglio et al. reported sensitivity of 63.2%, stressing the importance of the presence of the primary tumor at the time of blood testing, as they reported <50% sensitivity in patients with resected primary tumors [50]. In an attempt to improve sensitivity, they generated a new design of the panel with shorter amplicons, which only slightly increased sensitivity (68.4%) [50]. Bachet et al. and Jia et al. reported sensitivities for *RAS* mutation detection to be 76% and 93.3%, respectively [55,56]. Sensitivity for *KRAS* detection was 67% in Yao et al. [57], while it was 100% for the detection of all four genes tested by Wang et al. [66]. Only four of the studies that used NGS in tumor tissue reported sensitivity, which was 85% and 92% in Yao et al. and Shi et al., respectively [57,67]. The other two studies aimed to assess the molecular makeup of metastases, and sensitivity of ctDNA NGS to detect mutations found in the primary tumor and/or metastases was the only measure reported by both of them [54,59]. In the first study, this was as low as 39% and 55% for mutations found in the primary tumor and liver metastases, respectively [54], explained by the authors by the inclusion in their study of patients with oligometastatic disease and hence, a lower tumor load compared to other studies. However, using an enrichment assay, they managed to enhance the ctDNA detection of variants found in tissue to 80% [54]. Similarly, the second study reported a sensitivity of 64% for mutations found in liver metastases [59]. Due to the limited number of studies and the range of platforms used, we did not attempt any inter-study comparison. Overall, these data suggest that most ctDNA NGS platforms may come as high-sensitivity liquid biopsy alternatives.

Although not included in Table 4, a separate note should be made for the study by Strickler et al., which had the largest population among the reviewed studies, counting 1397 unique patient samples [64]. Since their aim was to describe the mutational landscape of cfDNA, the researchers did not directly compare plasma with matched tissue. Instead, they referred to three large-scale genomic databases (i.e., TCGA, NHS/HPFS, and GENIE). Their results showed a strong association of mutational prevalence in cfDNA and tumors (*R*^2^ = 0.95; *p* < 0.0001), with *TP53*, *APC*, *KRAS*, and *PIK3CA* being the genes most commonly mutated in all four cohorts. Mutations in *EGFR* were more prevalent in cfDNA, which could possibly be attributed to differences inherent to the cohorts themselves, such as the inclusion of more heavily pretreated patients in the tissue-based cohorts. The same study also offered insight into the clonality of mutations and described EGFR ectodomain mutations and other mechanisms of resistance to therapeutic blockade of the EGFR. It went on to highlight some limitations of cfDNA, such as the possibility of false positive results, as one cannot be certain whether a mutation detected in cfDNA is actually derived from the patient’s tumor or a hematopoietic clone that has, transiently or not, developed this mutation [64].

### 4.3. Comparison of Plasma NGS with Other Liquid Biopsy Techniques

Five studies reported the validation of ctDNA detection with a reference method such as digital PCR (dPCR) [54,59] or droplet dPCR (ddPCR) [50,53,61]. These methods are known to have sensitivity of 0.01% and are generally used to validate NGS results [8]. The superiority of digital PCR methods over NGS was demonstrated in most of these studies. In Furuki et al., it was reported to be 89% compared to only 64% for NGS [59]. Similarly, Demuth et al. found ddPCR to be more successful than NGS in identifying the *KRAS* mutations in ctDNA (in 100% versus 86% of cases, respectively), and to exhibit a higher concordance rate with tissue than NGS (89% versus 79%) [61]. Zhang et al. used ddPCR as reference to set the cutoff values for their NGS approach and had similar findings [53]. In the study of Beije et al., 93% of mutations observed in the primary and/or secondary tumor location(s) were detected with dPCR, leading the authors to recommend this method to track mutations previously detected with tissue NGS [54]. Finally, Rachiglio et al. subjected plasma *RAS*-negative cases with available material to ddPCR, which identified mutations in three out of five samples, at lower allelic frequencies than the 1% detection limit of NGS [50].

## 5. Conclusions

Given the latest ESMO guidelines for the molecular testing of mCRC, one would argue for the need for parallel testing of a gene panel including at least *KRAS* and *NRAS* exons 2, 3, and 4, and *BRAF* exon 15 [2]. Numerous assays have been developed for routine use in archival tissue samples that often contain poor-quality and low-in-quantity DNA. However, it is known that the mutation status of CRC may change among treatment lines. It seems that the continuum of care in mCRC will soon demand real-time analysis for at least the above-mentioned mutations, so as to accurately challenge patients with targeted agents at later lines of therapy. A number of strategy-testing clinical trials tackle this issue by serial liquid biopsies [73,74].

In view of the data reviewed herein, ctDNA analysis with NGS is feasible and exhibits satisfactory concordance rates with reference methods of molecular testing, such as dPCR, in tissue or plasma. With randomized clinical trial validation, this type of liquid biopsy may offer valuable insight into the tumor molecular signature at diagnosis of metastatic disease, as well as an accurate update of this information at disease progression. Similarly, the application of NGS panels in ctDNA would allow for characterization of emergent mutations and unveil therapy resistance. With its ability to test for multiplex genetic testing, plasma NGS could also be relevant as a screening tool for clinical trials with a molecular target. A few years ago, Overman et al. reported the use of tissue NGS in the context of the Assessment of Targeted Therapies Against CRC (ATTACC) trial [75]. This was an umbrella molecular screening program for patients with 5-fluorouracil-refractory mCRC. Based on these results, patients were assessed for eligibility for companion trials. Out of 454 patients enrolled, 95% had available tissue and a valid biomarker result and 32% were enrolled in a clinical trial (92 on biomarker-selective and 65 on non-biomarker-selective). In a similar clinical study where plasma NGS would be used, patients could be centrally screened for targetable genetic aberrations and, based on these results, subsequently referred for enrollment in a relevant study. It is plausible that the lower limit of detection of plasma NGS could yield an even higher amount of valid biomarker results and make such screening more broadly available to patients.

However, it seems that there is not enough data to support routine screening of mCRC patients with NGS panels that extend beyond currently actionable genes, outside of the context of clinical trials. When conducting such studies or interpreting their results, one should be aware of the variety of NGS platforms and gene panels available, as well as of the multitude of pre-analytical steps that should be taken and meticulously reported, which was found to be a frequent omission in the studies we reviewed. Despite the potential of ctDNA NGS in mCRC, one should not overlook its inherent limitations, which are accurately summarized in a review by Denis et al. [76]. In brief, it cannot compete with digital PCR in terms of sensitivity, and thus requires validation with such a method in every laboratory. The issue of sensitivity is highly relevant in the context of liquid biopsies, where mutant ctDNA is like the “needle in a haystack” of wild-type DNA fragments. NGS is also related to longer turnaround time and a higher risk of incidental findings, which can only worsen the situation of “false positive” mutations originating from hematopoietic clones rather than the tumor itself. Even NGS’ ability to detect additional gene mutations is often frowned upon by experts, on the basis of a lack of well-established clinical utility or alignment to therapeutic guidelines [77].

In the near future, it is expected that the development of NGS platforms with higher sensitivity will further upgrade the study of tumor heterogeneity in the blood, possibly at the level of single-cell sequencing. This knowledge of the genetic, epigenetic, and other -omic make-up of the tumor from the blood compartment may enhance the discrimination of oncogenic drivers that are critical for cancer cell survival and progression from passenger mutations. The use of targeted NGS to monitor these mutations in real time may set the foundation for a new approach in the management of cancer. Additionally, the progress of Molecular Oncology coupled with the emergence of an increasing number of actionable genetic aberrations make it highly probable that NGS will soon become a necessary tool for the design of combinatory targeted therapies. We are optimistic that with these advances, we are not far from turning truly personalized cancer treatment from a moving target into reality.

## Figures and Tables

**Table 1:** Overview of the studies using plasma next-generation sequencing for advanced colorectal cancer in the clinical setting.

First Author, Year of Publication	Study Population	Number of Advanced CRC Patients with Plasma NGS	Study Aim	Ref.
Beránek et al., 2016	mCRC patients	32	Comparison of cfDNA extraction methods	[58]
Furuki et al., 2018		22	Study of CRC heterogeneity	[59]
Hsu et al., 2018		32	Monitoring response to treatment	[60]
Demuth et al., 2018		28	Comparison of genotyping methods	[61]
Osumi et al., 2018		101	Assessment of feasibility and clinical relevance	[62]
Ghatalia et al., 2019		33	Assessment of feasibility and clinical relevance	[63]
Strickler et al., 2018	CRC patients, advanced	1397	Study of CRC heterogeneity	[64]
Kato et al., 2019	CRC patients, 96% mCRC	94	Monitoring response to treatment	[65]
Yamauchi et al., 2017	mCRC patients, bevacizumab-treated at first line	21	Study of the development of resistance	[52]
Peeters et al., 2018	mCRC patients, panitumumab-treated at first line (ASPECCT trial)	261	Study of the development of resistance	[48]
Zhang et al., 2019	mCRC, cetuximab-treated at first line	15	Comparison of genotyping methods	[53]
Khan et al., 2018	mCRC, RAS wild-type, chemotherapy-refractory (Prospect-C trial)	23	Study of the development of resistance	[44]
Beije et al., 2016	mCRC patients, resection of liver metastases	12	Comparison of genotyping methods	[54]
Tie et al., 2015	mCRC patients, chemotherapy-naïve	54	Monitoring response to treatment	[45]
Bachet et al., 2018		412	Comparison of genotyping methods	[55]
Yao et al., 2018		76	Monitoring response to treatment	[57]
Jia et al., 2019		41	Monitoring response to treatment	[56]
Rachiglio et al., 2016	Metastatic NSCLC and CRC patients (mCRC cohort)	35	Assessment of feasibility and clinical relevance	[50]
Kim et al., 2015	Solid tumor patients (mCRC cohort)	32	Assessment of feasibility and clinical relevance	[49]
Onidani et al., 2019	Head and neck cancer and gastrointestinal cancers (second phase with mCRC)	7	Study of the development of resistance	[51]
Wang et al., 2019	mCRC	184	Analytical validation and assessment of clinical relevance	[66]
Shi et al., 2019	Metastatic or locally advanced unresectable CRC	34	Assessment of feasibility and clinical relevance	[67]

CRC, colorectal cancer; mCRC, metastatic colorectal cancer; NGS, next-generation sequencing; cfDNA, cell-free DNA; NSCLC, non-small-cell lung cancer; Ref., reference.

**Table 2:** Summary of NGS platforms and gene panels used in the reviewed studies.

Ref.	Company	Platform	Gene Panel	Number of Tested Genes	Library Preparation Protocols
[60]	Life Technologies	Ion PGM	Custom panel	12	NR
[54]				21	Ion AmpliSeq Library Kit 2.0
[56]			SV-CA50-ctDNA panel	50	Ion AmpliSeq Library Kit 2.0
[59]			Ion AmpliSeq Cancer Hotspot panel v2	50	Ion AmpliSeq Kit
[51]			Ion AmpliSeq Cancer Hotspot panel v2	50	Ion AmpliSeq Library Kit Plus
[50]			Not reported	22	Oncomine Solid Tumor DNA Kit
[61]			Ion AmpliSeq Colon & Lung v2	22 (*KRAS*) *	Oncomine Solid Tumor DNA Kit
[55]		Ion Proton		22	NR
[62]		Ion S5	Custom panel	14	Oncomine Colon cfDNA
[45]	Illumina	MiSeq	Custom panel	15 (single mutation) *	NR
[58]			SOMATIC 1 MASTRTM v2	*BRAF*, *KRAS*, *NRAS*	NR
[66]		MiSeq	Accu-Act Panel	61	CRC01
[52]		HiSeq 2500	NCC Oncopanel	90	KAPA Hyper-prep
[57]			Custom assay	40 (*KRAS*, *NRAS*, *HRAS*, *BRAF*) *	SureSelect QTX
[44]		NextSeq 500	AVENIO ctDNA Expanded Kit	77	Custom
[53]			Custom assay	18	NR
[48]		NR	PlasmaSelect-R	63	NR
[49]	Guardant Health	Guardant360		54	Hybrid capture
[64]				54, 68, or 70	
[65]				54–73	
[63]				73	
[67]	Guardant Health	Guardant360	Ion AmpliSeq 2.0	68	
	Thermo Fisher Scientific	Ion Torrent	Oncomine Comprehensive Cancer Panel	143	

* In parentheses, the genes the status of which was of interest to the study. Ref, reference. NR, not reported.

**Table 3:** Pre-analytical and technical parameters.

Ref.	Whole Blood Input (mL)	Tubes	Time to Centrifugation	Centrifugation 1	Centrifugation 2	Plasma Input (mL)	DNA Input (ng)
[58]	9-10	EDTA	1 h	1300 rcf	12,000 rcf	0.75	0.35–4
[59]	NR	EDTA	3 h	1900 rcf	16,000 rcf	1	10
[60]	NR	Streck	NR	NR	-	2.5–4	NR
[61]	NR	NR	NR	2300	-	0.2–2	1.1–10
[62]	NR	EDTA	NR	1600 rcf	16,000 rcf	2	NR
[65]	10	NR	NR	NR	-	NR	NR
[52]	10	EDTA	Immediately	3500 rpm	12,000 rpm	3	40
[48]	5	EDTA	30 min	1500 rcf	-	NR	NR
[44]	NR	EDTA	1 h	1500 rcf	1500 rcf	4	25
[54]	30	EDTA	24 h	800 rcf	-	1	3–10
[45]	10	NR	3 h	NR	-	NR	3
[55]	30	Streck	Upon receival	1600 rcf	6000 rcf	NR	NR
[56]	NR	EDTA	NR	1600 rcf	16,000 rcf	NR	NR
[50]	10	EDTA	NR	1600 rcf	3000 rcf	2	10
[49]	NR	EDTA	Immediately	1600 rcf	-	1	NR
[51]	5	EDTA	24 h	NR	-	2	20
[66]	20	EDTA	2 h	1900 rcf	16,000 rcf	8	>20
[67]	20	Streck	NR	NR	-	2*1.4–1.8	6–20
[63]	20	Streck	NR	NR	-	NR	5–30
[53]	NR	NR	NR	NR	-	NR	2–60

Ref., reference; h, hour; min, minute; rcf, relative centrifugal force; rpm, revolutions per minute; NR, not reported.

**Table 4:** Summary of results generated with next-generation sequencing in plasma, in comparison with tissue and other liquid biopsy techniques.

Ref.	Prevalence of any Mutation	Prevalence of *RAS/RAF* Mutations in ctDNA	Method of Tissue Testing	Overall Tissue Concordance	*RAS/RAF* Tissue Concordance	Sensitivity of ctDNA NGS to Detect Known Tumor Tissue Variants	Cross-Platform Comparison
[61]	NR	NR	Routine PCR-based method	-	*KRAS*, 79%	NR	ddPCR
[62]	87.1%	*KRAS*, 38.6% *NRAS*, 4.9% *BRAF*, 7.9%		-	*RAS*, 77.2%	NR	-
[55]	73% (est.)	*KRAS*, 42% *NRAS*, 4%		-	*RAS*, 85.2%	*RAS*, 76%	-
[57]	NR	*KRAS*, 32.9%		-	*KRAS*, 81.25%	*KRAS*, 66.67%	-
[56]	95.7%	*KRAS*, 53.66% *NRAS*, 2.44% *BRAF*, 7.32%		-	*RAS*, 96%	*RAS*, 93.3%	-
[50]	NR	NR		-	NR	63.2%	ddPCR
[66]	*KRAS/NRAS BRAF/PIK3CA*, 40.76%	NR		79.89% (93.33% pre-treatment)	NR	100%	-
[49]	NR	NR	NGS	-	*KRAS*, 86.2% *BRAF*^V600E^, 100%	NR	-
[63]	NR	NR		-	*KRAS*, 66.67%	NR	-
[65]	79%	*KRAS*, 34%		-	*KRAS*, 75% *BRAF*^V600E^, 100%	NR	-
[45]	98.1%	NR		90.57% (est.)	NR	NR	-
[58]	NR	*KRAS*, 16% *NRAS*, 0% *BRAF*, 3%		86%	NR	NR	-
[60]	72%	*KRAS*, 41%		81%	NR	85%	-
[67]	NR	NR		85.29%	NR	92%	Digital sequencing
[54]	NR	NR		-	NR	*KRAS*, 39–55% (primary tumor-liver metastases)	OnTarget enrichment, dPCR
[59]	NR	NR		-	NR	64% (liver metastases)	dPCR

dPCR, digital polymerase chain reaction; (d)dPCR, (droplet) digital polymerase chain reaction; est., estimated.
